# Supplementary material for: Structure guided mimicry of an essential P. falciparum receptor-ligand complex enhances cross neutralizing antibodies
Source: Nat Commun. 2023 Sep 21;14:5879. doi: 10.1038/s41467-023-41636-5 (PMC10514071; doi:10.1038/s41467-023-41636-5)
Supplement: Supplementary file 1 — Supplementary Information [file 41467_2023_41636_MOESM1_ESM.pdf]

Supplementary Figure 1. (A) Sequence alignment of Fusion-F_{D12} and Fusion-R_{D12} chimeric proteins. The RON2L sequence in Fusion-F_{D12} and Fusion-R_{D12} are placed in the forward or reverse direction with respect to the AMA1 sequence and joined by linkers that connect them to the DII loop of AMA1. (B) Sequence alignment of HP41 and 3D7 AMA1-RON2L fusion proteins. Differences between the two proteins are highlighted red. The residues comprising the 1a, 1bcd, 1e, and 1f binding loops and the inserted RON2L sequence are highlighted.

Supplementary Figure 2. Biochemical and structural characterization of chimera Fusion-F_{D12}. (A) Coomassie stained SDS-PAGE gel of $AMA1_{D12}$ and Fusion-F_{D12} recombinant proteins. Red: reduced, NR: non-reduced. (B) Western blot analysis of $AMA1_{D12}$ and Fusion-F_{D12} proteins using conformation-dependent AMA1 mAbs 1F9 and 4G2 that bind AMA1 loop 1d and DII respectively. Removing DII loop of AMA1 to generate the fusion chimera negates mAb 4G2 binding while 1F9 epitope is still preserved. (C) Coomassie stained SDS-PAGE gel of $AMA1_{D12}$ and Fusion-R_{D12} recombinant proteins. Red: reduced, NR: non-reduced. (D) Western blot analysis of $AMA1_{D12}$ and Fusion-R_{D12} proteins using conformation-dependent AMA1 mAbs 1F9 and 4G2. Both antibodies did not bind Fusion-R_{D12}. (E) RON2L-Fc (1µg/mL) was used to test binding to recombinant $3D7AMA1_{D12}$, Fusion-$F_{D12HP41}$ and Fusion-$R_{D12HP41}$ proteins. $3D7AMA1_{D12}$ and Fusion-$R_{D12HP41}$ but not Fusion-$F_{D12HP41}$ bound to free RON2L. Data shown are mean±SEM of OD405 performed in three technical replicates. (F) RON2L-Fc binding to $3D7AMA1_{D12}$ but not Fusion-F_{D123D7}. Data shown are mean±SEM of OD405 performed in three technical replicates. (G) Right: Secondary structure view of Fusion-F_{D12} with boxes indicating the regions where the chimeric PfRON2L peptide begins and terminates. Left: Zoomed in orthogonal views of the PfAMA1-PfRON2L contacts showing the successful fusion of the

PfAMA1 with PfRON2L. Few residues of both PfAMA1 and PfRON2L are indicated in ball and sticks for clarity.

Supplementary Figure 3. Antibody titer in purified IgG or serum of apoAMA1, AMA1+RON2L binary complex and Fusion-F_{D12} immunized animals. (A) Antibody titer against 3D7AMA1, Fusion-F_{D12}, or FVOAMA1 antibody in the purified IgG from animals immunized with the indicated antigens in AddaVax and Freund's adjuvants. Data are shown for individual animals (n=4-5 per group) and each data point is the average of three replicates. Horizontal lines show the mean titer within each group. (B) Antibody titer against 3D7AMA1, Fusion-F_{D12}, or FVOAMA1 in the serum of animals immunized with the indicated antigens in AddaVax and Freund's adjuvants. Data are shown for individual animals (n=5 per group) and each data point is the average of three replicates. Horizontal lines show the mean titer within each group. (C, D) Anti-3D7 AMA1 titer in the purified IgG from the three independent immunization studies using Freunds or AddaVax adjuvants. Log10 transformed titer was used to compare IgG between apoAMA1 $_{D12}$ (C) and Fusion-F $_{D12}$ (D) immunized animals. Brown-Forsythe and Welch ANOVA test was performed to compare differences between groups. Horizontal lines show the mean antibody titer in each group. (E, F) Anti-3D7 AMA1 titer in the serum from two independent immunization studies using Freunds or AddaVax adjuvants. Log10 transformed titer was used to compare antibody titer between apoAMA1 $_{D12}$ (E) and Fusion-F_{D12} (F) immunized animals. Brown-Forsythe and Welch ANOVA test was performed to compare differences between groups. Horizontal lines show the mean antibody titer in each group.

Supplementary Figure 4: Antibody titer and neutralizing activity of purified IgG from apoAMA1, binary complex (AMA1+RON2L) and Fusion-F_{D12} immunized animals. (A) *In vitro* neutralization (1-cycle) assay against vaccine-type Pf3D7 parasites using 2mg/mL of total IgG from each animal. Data are shown for individual animals (n=4 per group) and each data point is the average of three replicates. Horizontal lines show the mean neutralizing activity in each group. Brown-Forsythe and Welch ANOVA test was performed to compare differences between groups. (B) AMA1-specific antibody titers in purified IgG from animals immunized with 3D7apoAMA1$_{D12}$ (blue square), 3D7AMA1$_{D12}$+RON2L binary complex (green circle), and Fusion-$F_{D12HP41}$ (purple triangle) immunized rats in Freund's adjuvant. Data are shown for individual animals (n=4 per group) and each data point is the average of three replicates. Horizontal lines show the mean antibody titer in each group. Brown-Forsythe and Welch ANOVA test was performed to compare differences between groups. (C) Proportion of IgG binding to non-vaccine type FVOAMA1 to vaccine-type 3D7AMA1. Data are shown for individual animals (n=4 per group) and each data point is the average of three replicates. Horizontal line marks the mean for each group. One-way ANOVA was performed to compare differences between groups.

Supplementary Figure 5. Parasite neutralization assay using IgG and serum. (A) Relationship between anti-AMA1 antibody titer in serum (x-axis) and neutralizing (2-cycle) activity in 2% serum (y-axis) between Fusion-F_{D12} (purple triangle) and apoAMA1$_{D12}$ (blue square) antigens in Freund's adjuvant. Each data point is the average of two replicates. (B) Left panel: proportion of IgG1 to IgG2a AMA1-specific antibodies in rat sera of Fusion-F_{D12} (purple) relative to apoAMA1$_{D12}$ (blue) immunized animals in the AddaVax groups. Data shown are

mean±SEM (n = 5 per group) performed in duplicate. Right panel: proportion of IgG1 to IgG2a Fusion-F_{D12}-specific antibodies in rat sera of Fusion-F_{D12} (purple) relative to apoAMA1$_{D12}$ (blue) immunized animals in the AddaVax group. Data are mean±SEM (n = 4 per group). Welch's t-test was performed to compare differences between groups. (C) Left panel: Proportion of IgG1 to IgG2a AMA1-specific antibodies in rat sera of Fusion-F_{D12} (purple) relative to apoAMA1$_{D12}$ (blue) immunized animals in the Freund's group. Data shown are mean±SEM (n = 5 per group). Right panel: Proportion of Fusion-F_{D12} vs apoAMA1$_{D12}$ specific IgM type antibodies in the sera of Fusion-F_{D12} (purple) relative to apoAMA1$_{D12}$ (blue) immunized animals in the Freund's group. Data are mean±SEM (n = 5 per group). Welch's t-test was performed to compare differences between groups. (D) Anti-RON2L-specific IgG titer in serum of apoAMA1$_{D12}$ (blue) or Fusion-F_{D12} (purple) antigen immunized animals in Freund's adjuvant. Data shown are mean±SEM (n = 5 per group. In all experiments the average of technical replicates for each animal was used for analysis. Welch's t-test was performed to compare differences between groups. (E) IgA titer in serum of apoAMA1$_{D12}$ (blue) or Fusion-F_{D12} (purple) antigen immunized animals in Freund's adjuvant. Data shown are mean±SEM (n = 5 per group. In all experiments the average of two technical replicates for each animal was used for analysis. Welch's t-test was performed to compare differences between groups.

Supplementary Figure 6. Antibody type and isotype differences between apoAMA1$_{D12}$ and Fusion-F_{D12} immunized rat serum and parasite neutralizing activity. (A) ELISA OD_{450} after AMA1 titer normalization of IgG from apoAMA1$_{D12}$ and Fusion-F_{D12} groups (n=5 per group) used in Fig 3B, Fig 4A-4C, 4F. (B) Comparison of 1-cycle vs 2-cycle parasite neutralization assay. Spearman's rank test (rS) was performed to analyze correlation between the two assays.

Suppl Fig 1

A

```
Fusion-FD12          NYMGNPWTEYMAKYDIEEVHGSGIRVDLGEDAEVAGTQYRLPSGKCPVFGKGIIIENSKT
Fusion-RD12          NYMGNPWTEYMAKYDIEEVHGSGIRVDLGEDAEVAGTQYRLPSGKCPVFGKGIIIENSKT

Fusion-FD12          TFLTPVATGNQYLKDGGFAFPPTEPLMSPMTLDDMRLLYKDNEDVKNLDELTLCSRHAGN
Fusion-RD12          TFLTPVATGNQYLKDGGFAFPPTEPLMSPMTLDDMRLLYKDNEDVKNLDELTLCSRHAGN

Fusion-FD12          MIPDNDKNSNYKYPAVYDDKDKKCHILYIAAQENNGPRYCNKDESKRNSMFCFRPAKDIS
Fusion-RD12          MIPDNDKNSNYKYPAVYDDKDKKCHILYIAAQENNGPRYCNKDESKRNSMFCFRPAKDIS

Fusion-FD12          FQNLVYLSKNVVHNWEKVCPRKNLQNAKFGLWVDGNCEDIPHVNEFSANDLFECNKLVFE
Fusion-RD12          FQNLVYLSKNVVHNWEKVCPRKNLQNAKFGLWVDGNCEDIPHVNEFSANDLFECNKLVFE

                                     RON2L (Forward construct)          Linker
Fusion-FD12          LSASDQPKQYEQHLTQQAKDIGAGPVASCFTTRMSPPQQICLNSVVNTALSGGSGGGNAA
Fusion-RD12          LSASDQPKQYEQHLGGSGG--GGGSLATNVVSNLCIQQPPSMRTTFCSAVPG------AG
                                   Linker       RON2L (Reverse construct)

Fusion-FD12          MIKSAFLPTGAFKADRYKSHGKGYNWGNYNTETQKCEIFNVKPTCLINDKNYIATTALSH
Fusion-RD12          IDKAQQGGSGGGGGDRYKSHGKGYNWGNYNTETQKCEIFNVKPTCLINDKNYIATTALSH
                           Linker

Fusion-FD12          PIEVEAAA
Fusion-RD12          PIEVEAAA
```

B

```
                                                                         1a
HP41-RON2L       -------------------NYMGNPWTEYMAKYDIEEVHGSGIRVDLGEDAEVAGTQYRL
3D7-RON2L        -------------------NYMGNPWTEYMAKYDIEEVHGSGIRVDLGEDAEVAGTQYRL

                                            1bcd loops
HP41-RON2L       PSGKCPVFGKGIIIENSKTTFLTPVATGNQYLKDGGFAFPPTEPLMSPMTLDDMRLLYKD
3D7-RON2L        PSGKCPVFGKGIIIENSKTTFLTPVATGNQYLKDGGFAFPPTEPLMSPMTLDEMRHFYKD

                                    1e loop
HP41-RON2L       NEDVKNLDELTLCSRHAGNMIPDNDKNSNYKYPAVYDDKDKKCHILYIAAQENNGPRYCN
3D7-RON2L        NKYVKNLDELTLCSRHAGNMIPDNDKNSNYKYPAVYDDKDKKCHILYIAAQENNGPRYCN

                   1f loop
HP41-RON2L       KDESKRNSMFCFRPAKDISFQNLVYLSKNVVHNWEKVCPRKNLQNAKFGLWVDGNCEDIP
3D7-RON2L        KDESKRNSMFCFRPAKDISFQNLVYLSKNVVDNWEKVCPRKNLQNAKFGLWVDGNCEDIP

                                                                 RON2L
HP41-RON2L       HVNEFSANDLFECNKLVFELSASDQPKQYEQHLTQQAKDIGAGPVASCFTTRMSPPQQIC
3D7-RON2L        HVNEFPAIDLFECNKLVFELSASDQPKQYEQHLTQQAKDIGAGPVASCFTTRMSPPQQIC

                   RON2L   Linker
HP41-RON2L       LNSVVNTALSGGSGGGNAAMIKSAFLPTGAFKADRYKSHGKGYNWGNYNTETQKCEIFNV
3D7-RON2L        LNSVVNTALSGGSGGGNASMIKSAFLPTGAFKADRYKSHGKGYNWGNYNTETQKCEIFNV

HP41-RON2L       KPTCLINDKNYIATTALSHPIEVE------------------------------------
3D7-RON2L        KPTCLINDKNYIATTALSHPIEVE------------------------------------
```

Suppl Fig 2

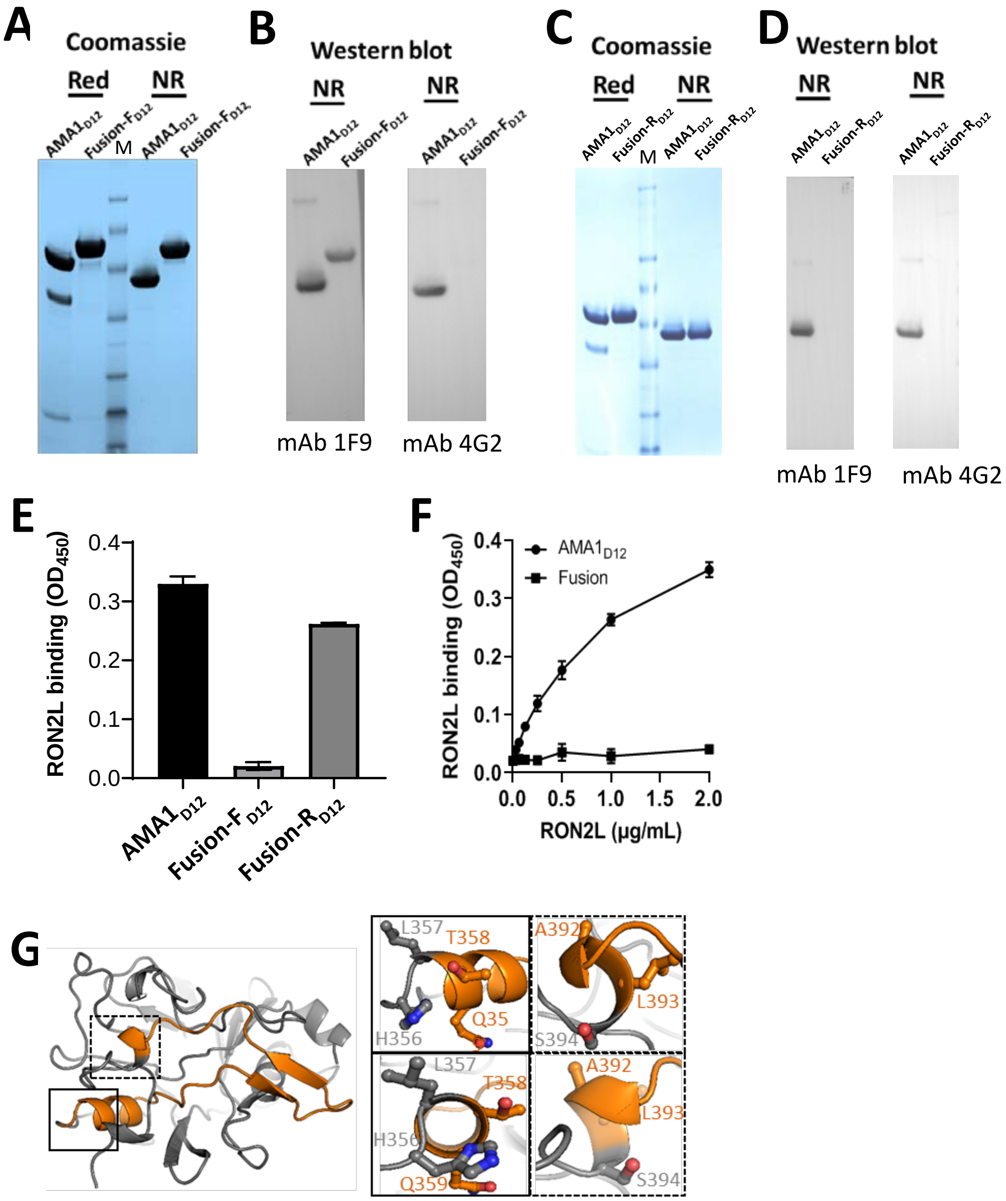

A
Coomassie
Red
NR
AMA1D12
Fusion-FD12
M
AMA1D12
Fusion-FD12
B
Western blot
NR
NR
AMA1D12
Fusion-FD12
AMA1D12
Fusion-FD12
mAb 1F9
mAb 4G2
C
Coomassie
Red
NR
AMA1D12
Fusion-RD12
M
AMA1D12
Fusion-RD12
D
Western blot
NR
NR
AMA1D12
Fusion-RD12
AMA1D12
Fusion-RD12
mAb 1F9
mAb 4G2
E
RON2L binding (OD450)
0.4
0.3
0.2
0.1
0.0
AMA1D12
Fusion-F D12
Fusion-RD12
F
RON2L binding (OD450)
AMA1D12
Fusion
0.4
0.3
0.2
0.1
0.0
0.0
0.5
1.0
1.5
2.0
RON2L (µg/mL)
G
L357
T358
Q35
H356
A392
L393
S394
L357
T358
H356
Q359
A392
L393
S394

Suppl Fig 3

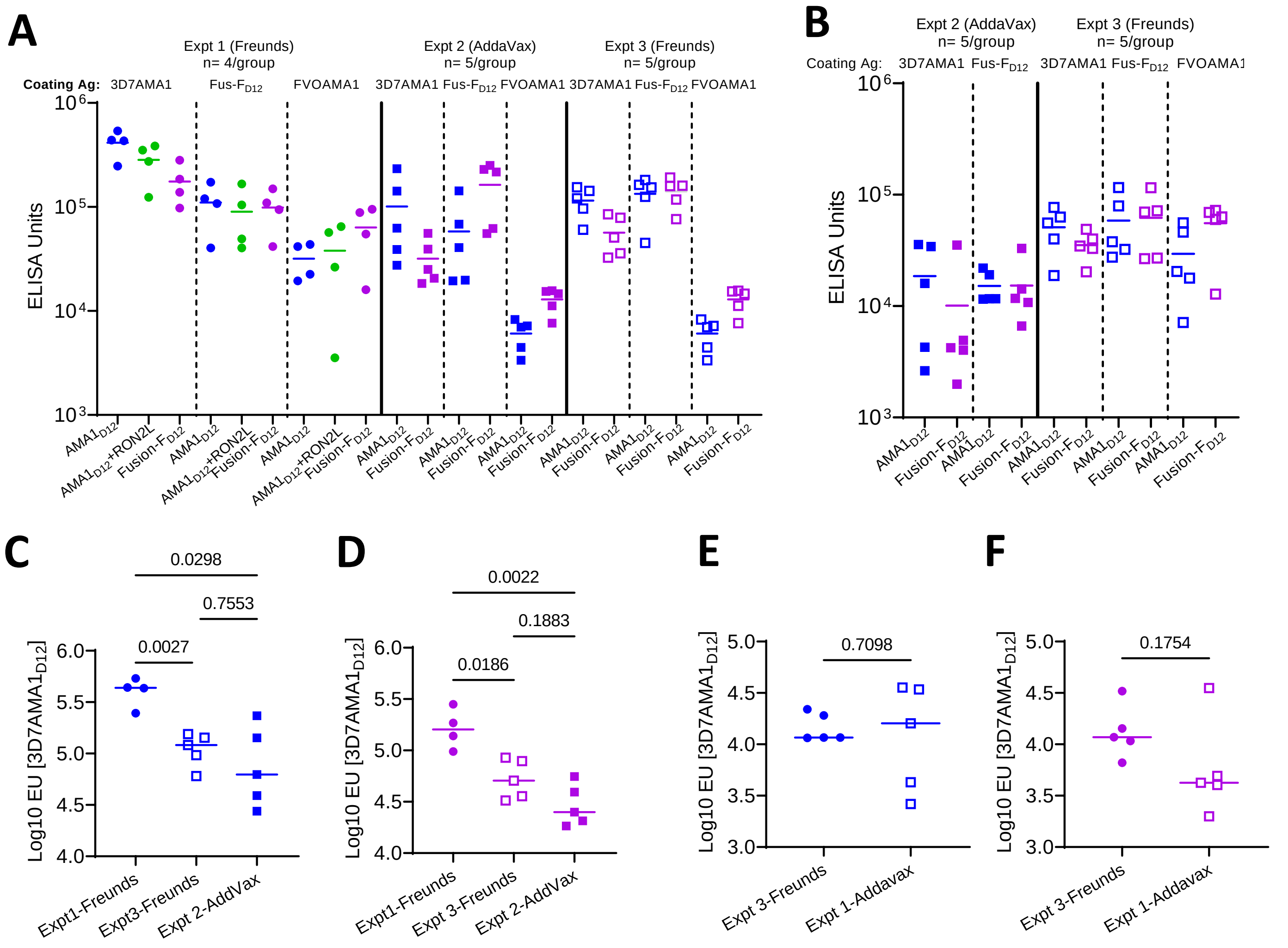
A
Expt 1 (Freunds)
n= 4/group
Expt 2 (AddaVax)
n= 5/group
Expt 3 (Freunds)
n= 5/group
Coating Ag: 3D7AMA1 Fus-FD12 FVOAMA1 3D7AMA1 Fus-FD12 FVOAMA1 3D7AMA1 Fus-FD12 FVOAMA1
ELISA Units
AMA1D12
AMA1D12+RON2L
Fusion-FD12
B
Expt 2 (AddaVax)
n= 5/group
Expt 3 (Freunds)
n= 5/group
Coating Ag: 3D7AMA1 Fus-FD12 3D7AMA1 Fus-FD12 FVOAMA1
ELISA Units
C
0.0298
0.7553
0.0027
Log10 EU [3D7AMA1D12]
Expt1-Freunds
Expt3-Freunds
Expt 2-AddVax
D
0.0022
0.1883
0.0186
Log10 EU [3D7AMA1D12]
Expt1-Freunds
Expt 3-Freunds
Expt 2-AddVax
E
0.7098
Log10 EU [3D7AMA1D12]
Expt 3-Freunds
Expt 1-Addavax
F
0.1754
Log10 EU [3D7AMA1D12]
Expt 3-Freunds
Expt 1-Addavax

Suppl Fig 4

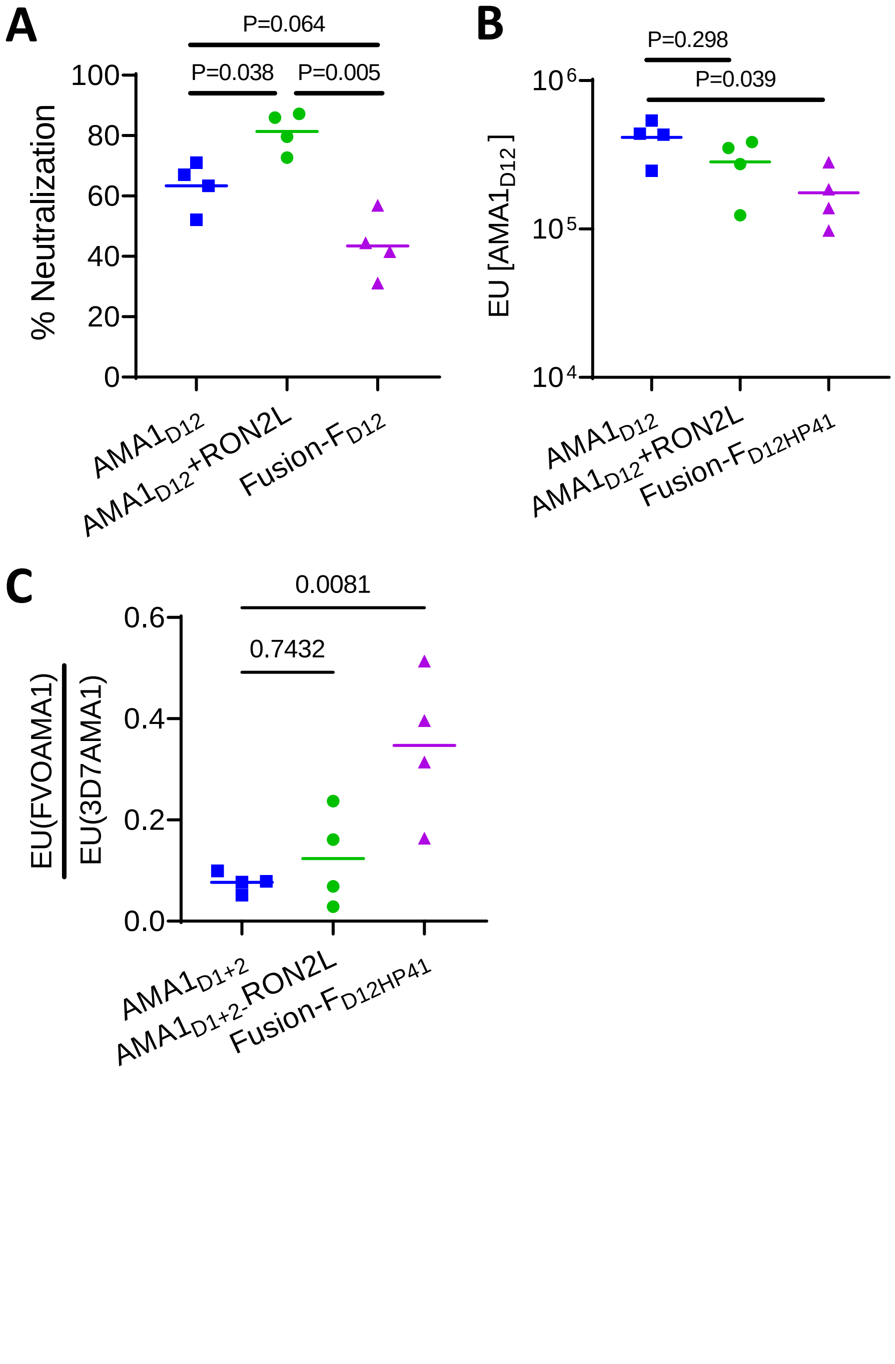
A
P=0.064
P=0.038
P=0.005
100
80
60
40
20
0
% Neutralization
AMA1D12
AMA1D12+RON2L
Fusion-FD12
B
P=0.298
P=0.039
10^6
10^5
10^4
EU [AMA1D12]
AMA1D12
AMA1D12+RON2L
Fusion-FD12HP41
C
0.0081
0.7432
0.6
0.4
0.2
0.0
EU(FVOAMA1)
EU(3D7AMA1)
AMA1D1+2
AMA1D1+2-RON2L
Fusion-FD12HP41

Suppl Fig 5

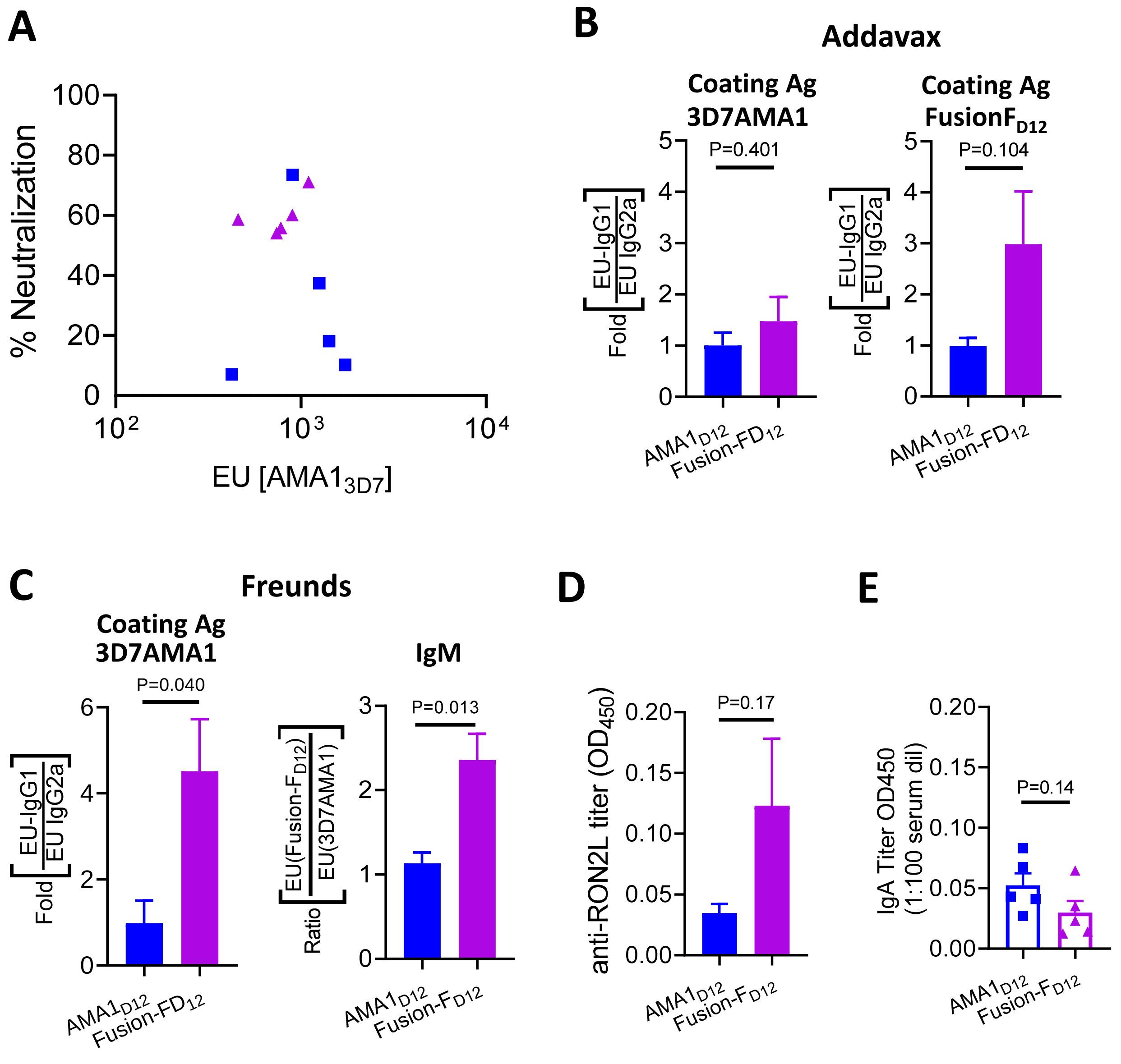

A
% Neutralization
100
80
60
40
20
0
10^2
10^3
10^4
EU [AMA1$_{3D7}$]
B
Addavax
Coating Ag 3D7AMA1
Coating Ag FusionF$_{D12}$
P=0.401
P=0.104
Fold [EU-IgG1 / EU IgG2a]
AMA1$_{D12}$
Fusion-FD$_{12}$
C
Freunds
Coating Ag 3D7AMA1
IgM
P=0.040
P=0.013
Fold [EU-IgG1 / EU IgG2a]
Ratio [EU(Fusion-F$_{D12}$) / EU(3D7AMA1)]
D
anti-RON2L titer (OD$_{450}$)
P=0.17
E
IgA Titer OD450 (1:100 serum dil)
P=0.14
AMA1$_{D12}$
Fusion-F$_{D12}$

Suppl Fig 6

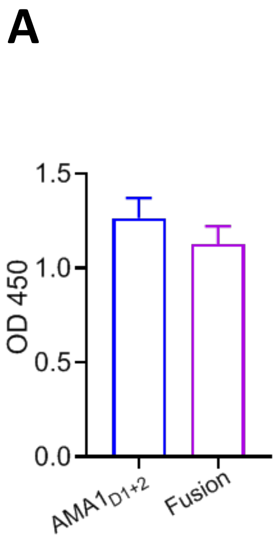
A
OD 450
1.5
1.0
0.5
0.0
AMA1D1+2
Fusion

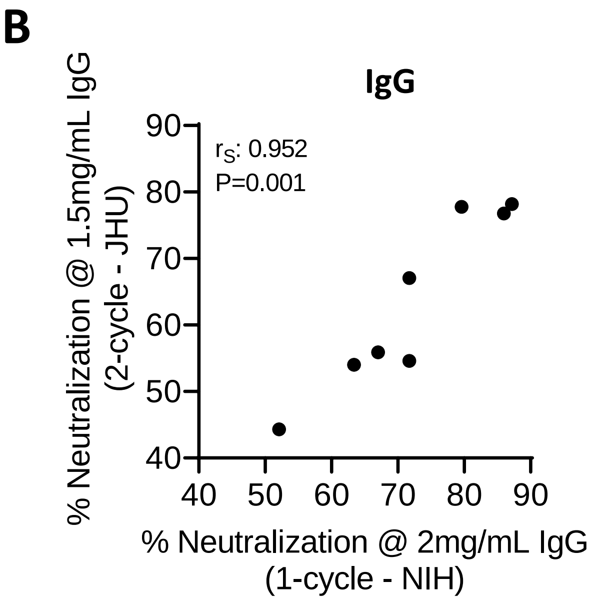
B
IgG
rS: 0.952
P=0.001
% Neutralization @ 1.5mg/mL IgG (2-cycle - JHU)
90
80
70
60
50
40
% Neutralization @ 2mg/mL IgG (1-cycle - NIH)

Supplementary Table 1. Data collection and refinement statistics

	Fusion-F_{D12}
A. Data collection statistics	
Spacegroup	$P3_12$
a, b, c (Å)	92.50, 92.50, 173.68
α, β, γ (deg.)	90, 90, 120
Wavelength	0.984
Resolution range (Å)	58.88 – 1.55 (1.58 – 1.55)
Measured reflections	855,225 (38,498)
Unique reflections	124,774 (6,114)
Redundancy	6.9 (6.3)
Completeness (%)	99.7 (100.0)
$I/\sigma(I)$	11.4 (2.1)
R_{merge}	0.075 (0.624)
B. Refinement statistics	
Resolution (Å)	57.89 – 1.55
R_{work} / R_{free}	0.190/0.219
No. of atoms	
Protein (A/B)	2703/2791
Solvent/Sulfate	700/5
Average B-values (Å^2)	
Protein (A/B)	25.5/28.9
Solvent/Sulfate	36.4/24.6
r.m.s. deviation from ideality	
Bond lengths (Å)	0.008
Bond angles (deg.)	1.14
Ramachandran statistics (%)	
Most favoured	98.0
Allowed	2.0
Disallowed	0.0

Values in parentheses are for the highest resolution shell
