# Supplementary material for: Structure guided mimicry of an essential P. falciparum receptor-ligand complex enhances cross neutralizing antibodies
Source: Nat Commun. 2023 Sep 21;14:5879. doi: 10.1038/s41467-023-41636-5 (PMC10514071; doi:10.1038/s41467-023-41636-5)
Supplement: Supplementary file 3 — Reporting Summary [file 41467_2023_41636_MOESM3_ESM.pdf]

nature portfolio

Corresponding author(s): Prakash Srinivasan

Last updated by author(s): Sep 5, 2023

Reporting Summary

Nature Portfolio wishes to improve the reproducibility of the work that we publish. This form provides structure for consistency and transparency in reporting. For further information on Nature Portfolio policies, see our Editorial Policies and the Editorial Policy Checklist.

Statistics

For all statistical analyses, confirm that the following items are present in the figure legend, table legend, main text, or Methods section.

n/a	Confirmed	
☐	☒	The exact sample size (*n*) for each experimental group/condition, given as a discrete number and unit of measurement
☐	☒	A statement on whether measurements were taken from distinct samples or whether the same sample was measured repeatedly
☐	☒	The statistical test(s) used AND whether they are one- or two-sided<br>*Only common tests should be described solely by name; describe more complex techniques in the Methods section.*
☒	☐	A description of all covariates tested
☐	☒	A description of any assumptions or corrections, such as tests of normality and adjustment for multiple comparisons
☐	☒	A full description of the statistical parameters including central tendency (e.g. means) or other basic estimates (e.g. regression coefficient) AND variation (e.g. standard deviation) or associated estimates of uncertainty (e.g. confidence intervals)
☐	☒	For null hypothesis testing, the test statistic (e.g. *F*, *t*, *r*) with confidence intervals, effect sizes, degrees of freedom and *P* value noted<br>*Give P values as exact values whenever suitable.*
☒	☐	For Bayesian analysis, information on the choice of priors and Markov chain Monte Carlo settings
☒	☐	For hierarchical and complex designs, identification of the appropriate level for tests and full reporting of outcomes
☒	☐	Estimates of effect sizes (e.g. Cohen's *d*, Pearson's *r*), indicating how they were calculated

Our web collection on statistics for biologists contains articles on many of the points above.

Software and code

Policy information about availability of computer code

Data collection	BMGLabtech MARS software was used to collect ELISA data, AttuneNXT v5.1.1 was used to collect parasitemia data.
Data analysis	GraphPad Prsim v.9.5.1

For manuscripts utilizing custom algorithms or software that are central to the research but not yet described in published literature, software must be made available to editors and reviewers. We strongly encourage code deposition in a community repository (e.g. GitHub). See the Nature Portfolio guidelines for submitting code & software for further information.

Data

Policy information about availability of data

All manuscripts must include a data availability statement. This statement should provide the following information, where applicable:

- Accession codes, unique identifiers, or web links for publicly available datasets
- A description of any restrictions on data availability
- For clinical datasets or third party data, please ensure that the statement adheres to our policy

All data associated with this paper are present in the main manuscript or in the Supplementary Materials. Atomic coordinates and structure factors have been deposited in the Protein Data Bank with PDB ID 8G6B. Source data are provided with the paper.

Research involving human participants, their data, or biological material

Policy information about studies with human participants or human data. See also policy information about sex, gender (identity/presentation), and sexual orientation and race, ethnicity and racism.

Reporting on sex and gender	*Use the terms sex (biological attribute) and gender (shaped by social and cultural circumstances) carefully in order to avoid confusing both terms. Indicate if findings apply to only one sex or gender; describe whether sex and gender were considered in study design; whether sex and/or gender was determined based on self-reporting or assigned and methods used. Provide in the source data disaggregated sex and gender data, where this information has been collected, and if consent has been obtained for sharing of individual-level data; provide overall numbers in this Reporting Summary. Please state if this information has not been collected. Report sex- and gender-based analyses where performed, justify reasons for lack of sex- and gender-based analysis.*
Reporting on race, ethnicity, or other socially relevant groupings	*Please specify the socially constructed or socially relevant categorization variable(s) used in your manuscript and explain why they were used. Please note that such variables should not be used as proxies for other socially constructed/relevant variables (for example, race/ethnicity should not be used as a proxy for socioeconomic status). Provide clear definitions of the relevant terms used, how they were provided (by the participants/respondents, the researchers, or third parties), and the method(s) used to classify people into the different categories (e.g. self-report, census or administrative data, social media data, etc.) Please provide details about how you controlled for confounding variables in your analyses.*
Population characteristics	*Describe the covariate-relevant population characteristics of the human research participants (e.g. age, genotypic information, past and current diagnosis and treatment categories). If you filled out the behavioural & social sciences study design questions and have nothing to add here, write "See above."*
Recruitment	*Describe how participants were recruited. Outline any potential self-selection bias or other biases that may be present and how these are likely to impact results.*
Ethics oversight	*Identify the organization(s) that approved the study protocol.*

Note that full information on the approval of the study protocol must also be provided in the manuscript.

Field-specific reporting

Please select the one below that is the best fit for your research. If you are not sure, read the appropriate sections before making your selection.

☒ Life sciences ☐ Behavioural & social sciences ☐ Ecological, evolutionary & environmental sciences

For a reference copy of the document with all sections, see nature.com/documents/nr-reporting-summary-flat.pdf

Life sciences study design

All studies must disclose on these points even when the disclosure is negative.

Sample size	Sample size calculation were not used due to the exploratory nature of this study. The immunization studies were conducted in 3 biolgical replicates with each study having 4-5 animals per group for a total of 14 animals per group. Sample size calculation is not applicable to the structural studies reported in the manuscript.
Data exclusions	none
Replication	The number of biological and technical replicates for each assay are shown in related figure legends and methods.
Randomization	Animals were randomized into groups before start of immunization. Randomization is not applicable for structural studies since only the fusion protein was crystallized and parasite neutralization assays as all samples from each group were evaluated
Blinding	Samples were blinded for assays performed at the GIA reference center, National Institutes of Health. ELISA experiments were conducted unblinded.

Reporting for specific materials, systems and methods

We require information from authors about some types of materials, experimental systems and methods used in many studies. Here, indicate whether each material, system or method listed is relevant to your study. If you are not sure if a list item applies to your research, read the appropriate section before selecting a response.

Materials & experimental systems

n/a	Involved in the study
☐	☒ Antibodies
☐	☒ Eukaryotic cell lines
☒	☐ Palaeontology and archaeology
☐	☒ Animals and other organisms
☒	☐ Clinical data
☒	☐ Dual use research of concern
☒	☐ Plants

Methods

n/a	Involved in the study
☒	☐ ChIP-seq
☐	☒ Flow cytometry
☒	☐ MRI-based neuroimaging

Antibodies

Antibodies used

Monoclonal antibodies 1F9 (gift from Dr. Robin Anders, La Trobe University) and 4G2 (gift from Dr. David Narum, NIH). Anti-rat IgG (Cat# 612-1325, Rockland), IgG1 (Cat# PA1-84708, Thermofisher Scientific), IgG2a (Cat# PA1-84709, Thermofisher Scientific) and IgM (Cat # 31476, Thermosisher Scientific) secondary antibody conjugated to HRP and mouse mAb HIS.H8 to 6x-His tag (Cat #MA1-21315, Thermofisher Scientific).

Validation

Antibodies 1F9 and 4G2 are validated and described in references 19 and 25 respectively

Eukaryotic cell lines

Policy information about cell lines and Sex and Gender in Research

Cell line source(s)

Sf9 cells were purchased from Thermo Scientific

Authentication

Sf9 cells were authenticated for viability and mycoplasma by Thermofischer (qPCR assay)

Mycoplasma contamination

Not detected

Commonly misidentified lines
(See ICLAC register)

none

Animals and other research organisms

Policy information about studies involving animals; ARRIVE guidelines recommended for reporting animal research, and Sex and Gender in Research

Laboratory animals

Rattus norvegicus, Sprague Dawley, Female 5-6 week old at the start of the experiment were obtained from Charles River Laboratories

Wild animals

no wild animals were used in this study

Reporting on sex

Female rats were used as previous studies did not find any sex specific differences in antibody titer to recombinant AMA1 vaccines. Furthermore, the studies tested activity of antibodies only in in vitro neutralization assays.

Field-collected samples

no field collected samples were used in this study

Ethics oversight

Johns Hopkins Animal Care and Use Committee (ACUC), under protocol RA22H291

Note that full information on the approval of the study protocol must also be provided in the manuscript.

Flow Cytometry

Plots

Confirm that:

☐ The axis labels state the marker and fluorochrome used (e.g. CD4-FITC).

☐ The axis scales are clearly visible. Include numbers along axes only for bottom left plot of group (a 'group' is an analysis of identical markers).

☐ All plots are contour plots with outliers or pseudocolor plots.

☐ A numerical value for number of cells or percentage (with statistics) is provided.

Methodology

Sample preparation

Describe the sample preparation, detailing the biological source of the cells and any tissue processing steps used.

Instrument

Identify the instrument used for data collection, specifying make and model number.

Software

Describe the software used to collect and analyze the flow cytometry data. For custom code that has been deposited into a community repository, provide accession details.

Cell population abundance

Describe the abundance of the relevant cell populations within post-sort fractions, providing details on the purity of the samples and how it was determined.

Gating strategy

Describe the gating strategy used for all relevant experiments, specifying the preliminary FSC/SSC gates of the starting cell population, indicating where boundaries between "positive" and "negative" staining cell populations are defined.

☐ Tick this box to confirm that a figure exemplifying the gating strategy is provided in the Supplementary Information.
